# Supplementary material for: Sequential metamaterials with alternating Poisson’s ratios
Source: Nat Commun. 2022 Feb 24;13:1041. doi: 10.1038/s41467-022-28696-9 (PMC8873317; doi:10.1038/s41467-022-28696-9)
Supplement: Supplementary file 1 — Supplementary Information [file 41467_2022_28696_MOESM1_ESM.pdf]

Sequential Metamaterials with Alternating Poisson's Ratios

Farzaneh et al.

Supplementary Information

Sequential Metamaterials with Alternating Poisson's Ratios

Amin Farzaneh[1], Nikhil Pawar[1], Carlos M. Portela[2], and Jonathan B. Hopkins[1*]

[1]Mechanical and Aerospace Engineering, University of California, Los Angeles, Los Angeles, CA 90095 USA
[2]Mechanical Engineering, Massachusetts Institute of Technology, Cambridge, MA 02139 USA
[*]Corresponding author, Email: hopkins@seas.ucla.edu

Supplementary Figures

The supplementary figures referenced in the main text along with their corresponding legends are provided on the following pages.

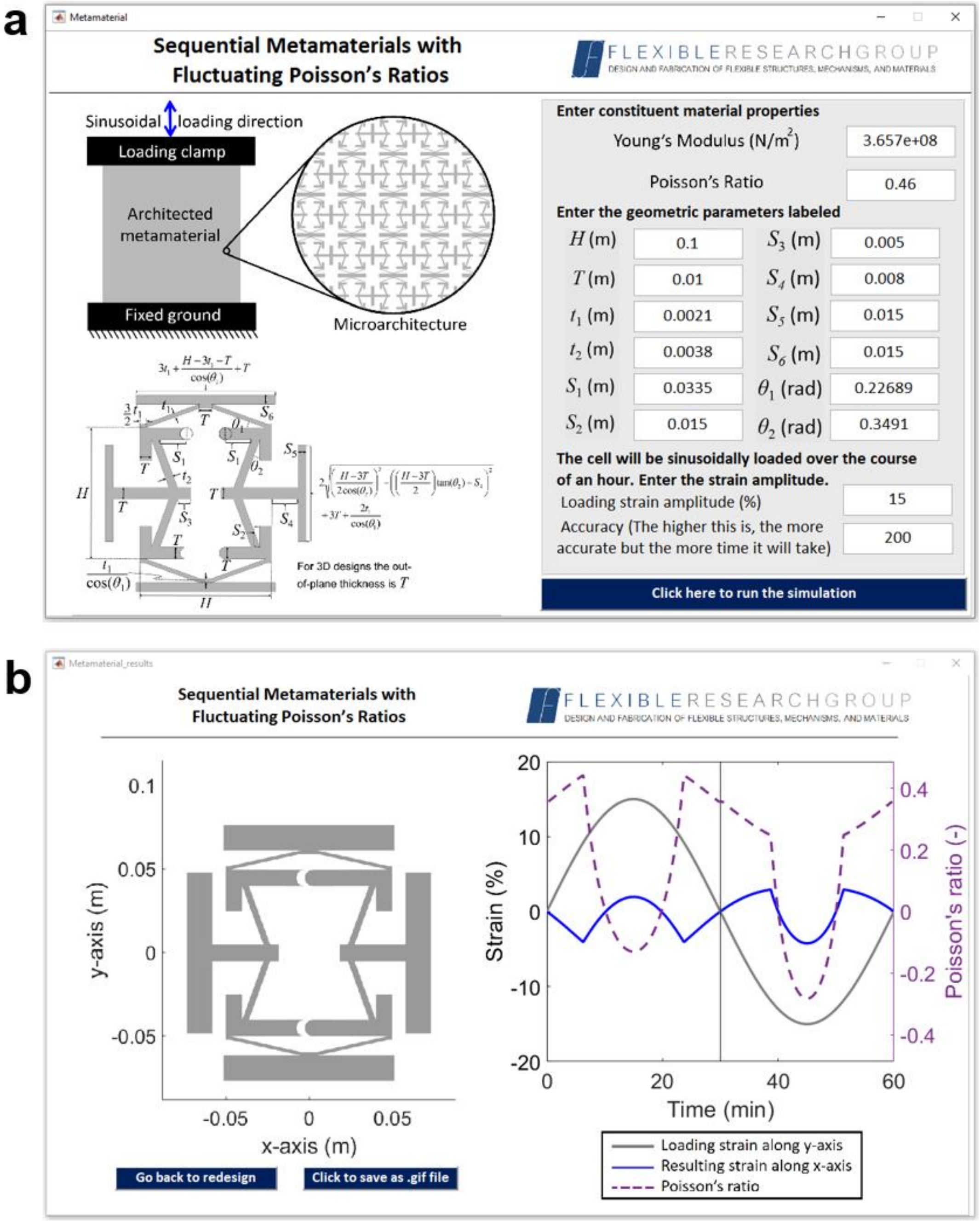

Supplementary Fig. 1 Graphical user interface (GUI) for the MATLAB-based metamaterial design tool. **a** When the tool launches, a screen requests users to specify metamaterial design parameters. **b** The tool then produces an animated version of the resulting design's unit cell being deformed next to a plot showing the resulting x-axis strain and Poisson's-ratio behavior in response to a sinusoidal y-axis strain input.

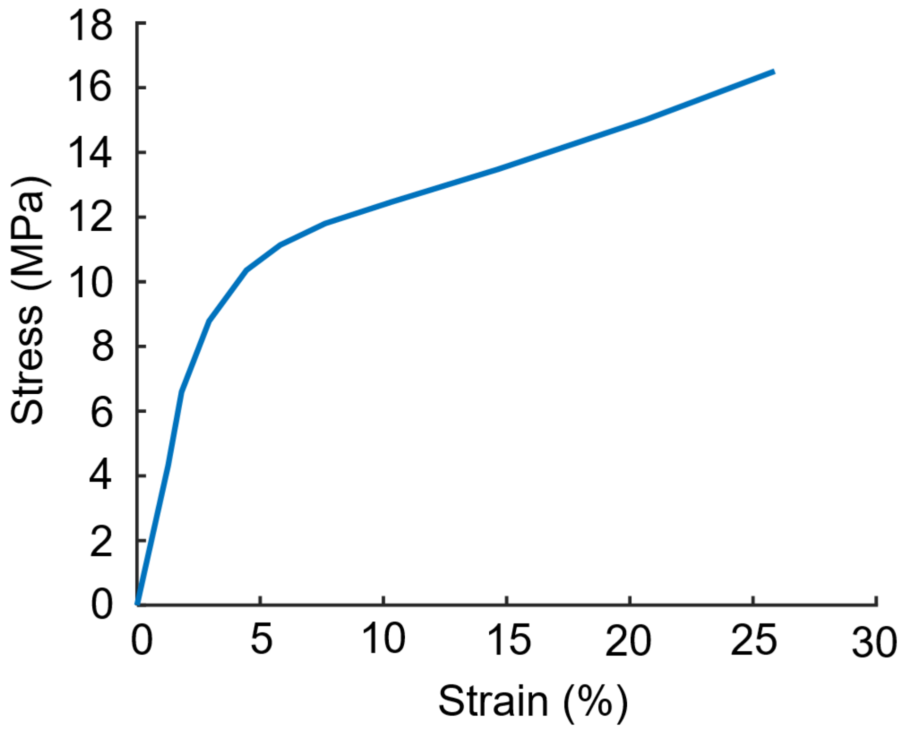

Supplementary Fig. 2 Stress-strain plot for Teflon used to model the nonlinear elastic-plastic behavior of the unit cell designs within the finite element analysis (FEA) plastic simulations performed in the main text.

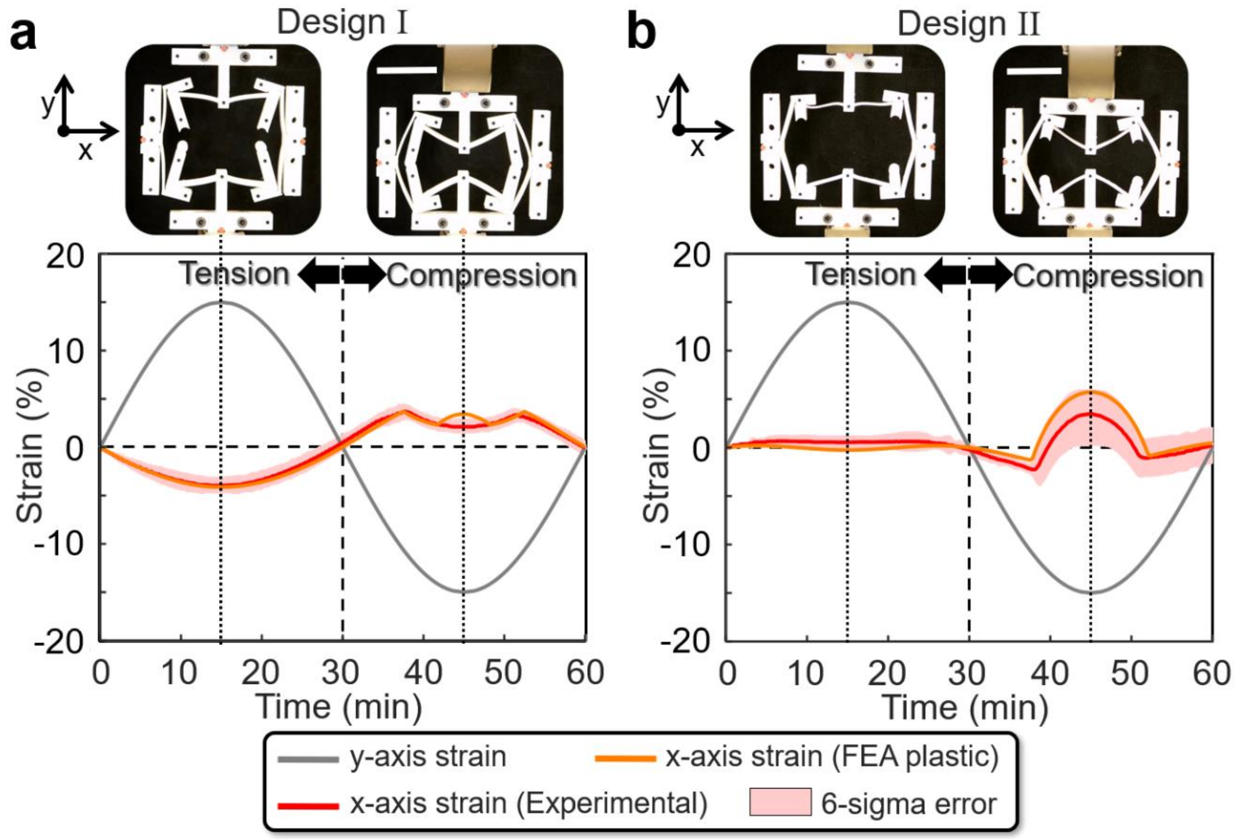

Supplementary Fig. 3 Plots demonstrating that cell designs turned 90° on their side achieve different Poisson's ratios than what the same designs achieve when they are loaded in their original orientation (compare with Fig. 5). **a** The x-axis strain response of Design I when it is turned 90° on its side and loaded sinusoidally along the y-axis. **b** The x-axis strain response of Design II when it is turned 90° on its side and loaded sinusoidally along the y-axis. Scale bars in **a** and **b**, 5 cm.

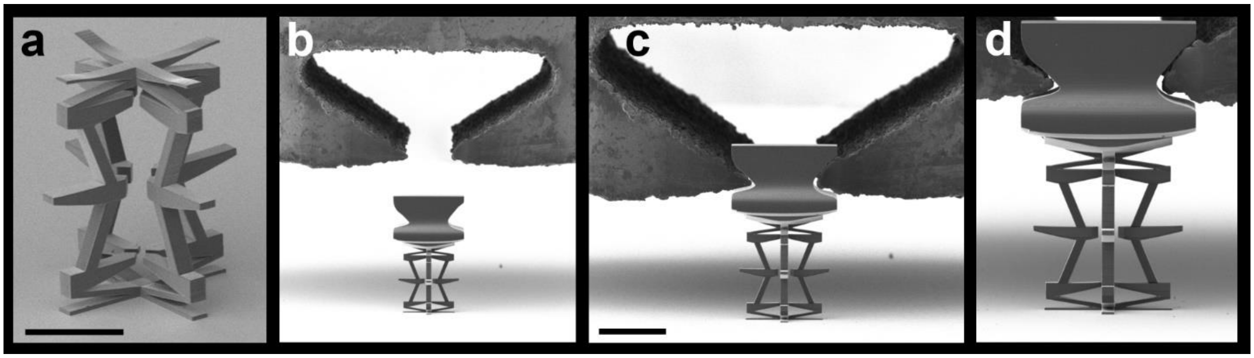

Supplementary Fig. 4 Scanning electron microscope (SEM) images of a 3D micro-scale unit cell that has been additively fabricated using two-photon lithography. **a** The cell shown without a tension-compression grip printed on its top tab. **b**, **c**, and **d** The cell shown at different scales with a tension-compression grip printed on its top tab. The nanoindenter's custom tension tip, which mates with this grip, is also shown. Scale bar in **a**, 50 µm, and in **c**, 100 µm.

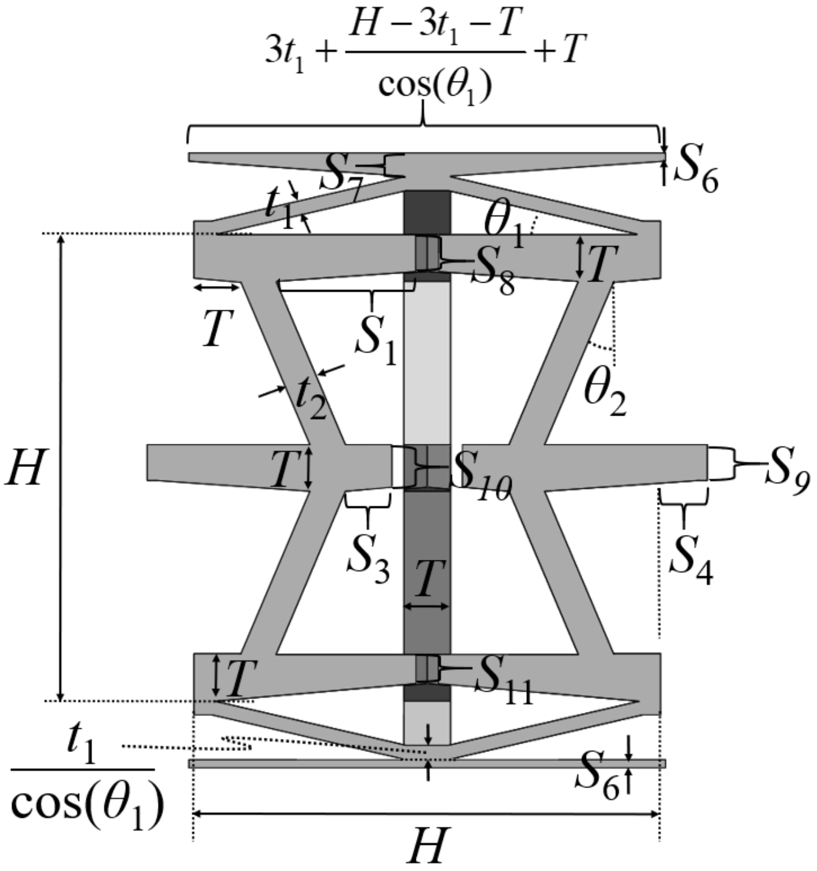

Supplementary Fig. 5 Geometric parameters that define the additively fabricated micro-scale 3D unit cell shown in Fig. 8 and Supplementary Fig. 4. The parameters are the same as those shown in Fig. 2 but new geometric parameters have been included to account for the features that needed to be tapered to enable the cell's additive fabrication.

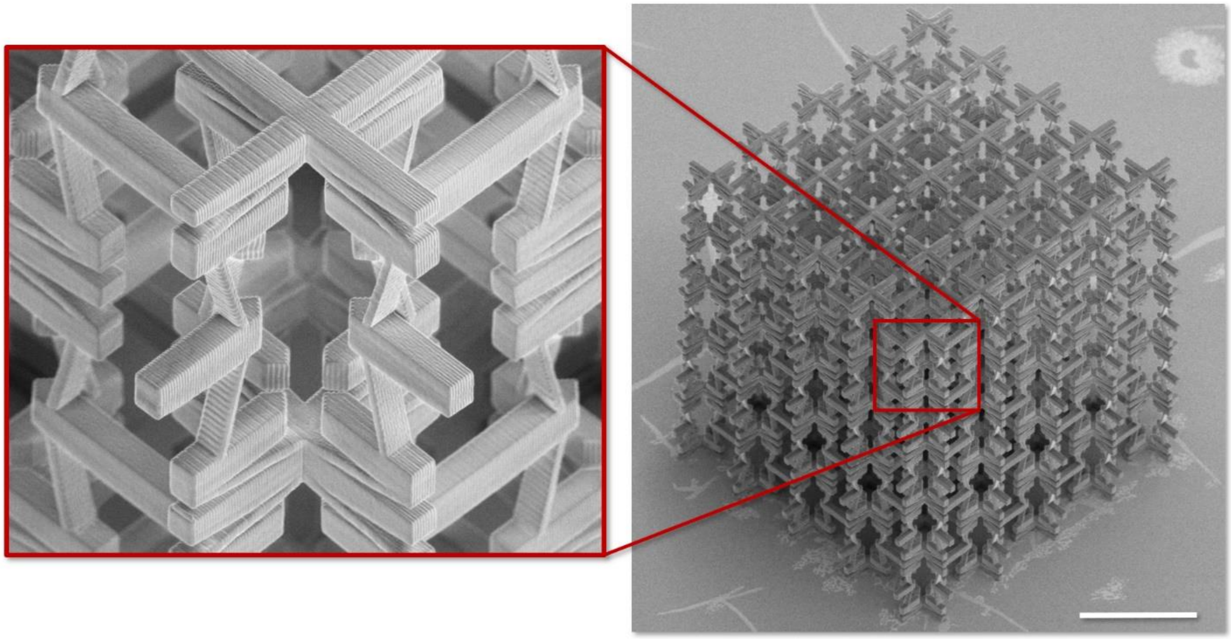

Supplementary Fig. 6 A 5x5x5 micro-scale lattice of unit cells that were additively fabricated using two-photon lithography. Scale bar, 50 μm.

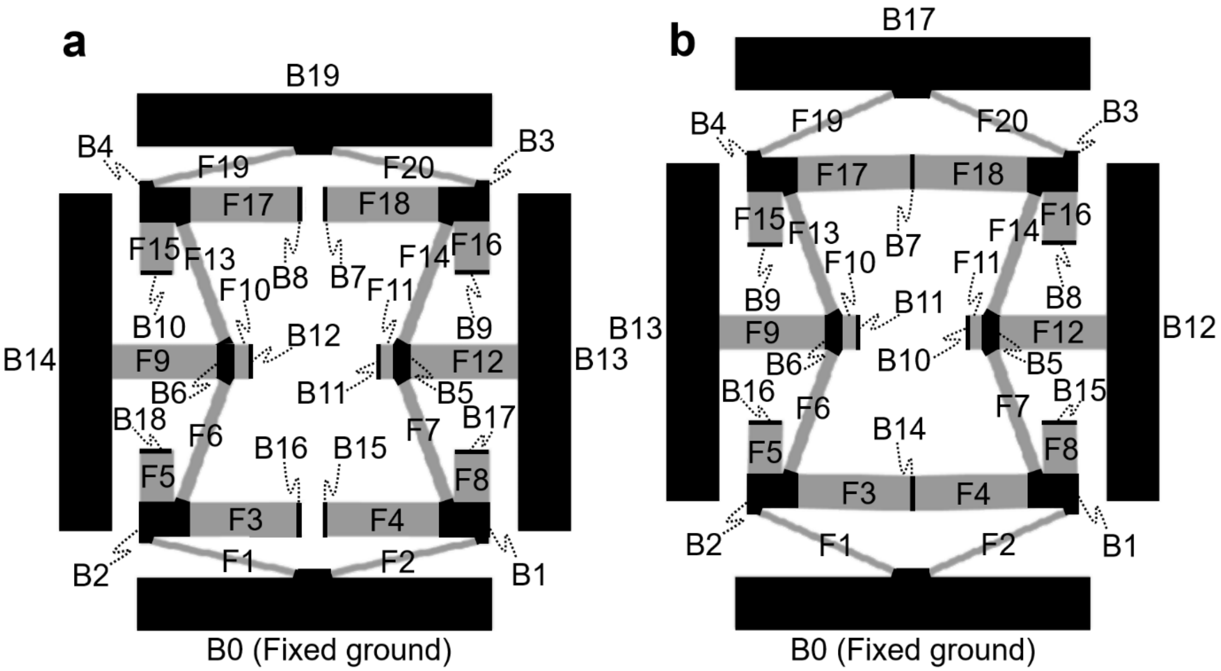

Supplementary Fig. 7 Graphical depiction explaining how the unit cell is analytically modeled by the MATLAB tool. **a** The numbered grey rectangles, labeled *F*, represent Bernoulli-Euler flexure beams and the numbered black shapes, labeled *B*, represent rigid bodies. *B0* is always the grounded body that is held fixed. **b** If bodies touch, e.g., bodies *B7* and *B8* or *B15* and *B16* in **a**, they are fused together by relabeling them as the same body number, e.g., bodies *B7* and *B14*, when constructing the cell's twist-wrench stiffness matrix. Note that all the other bodies must also be relabeled.

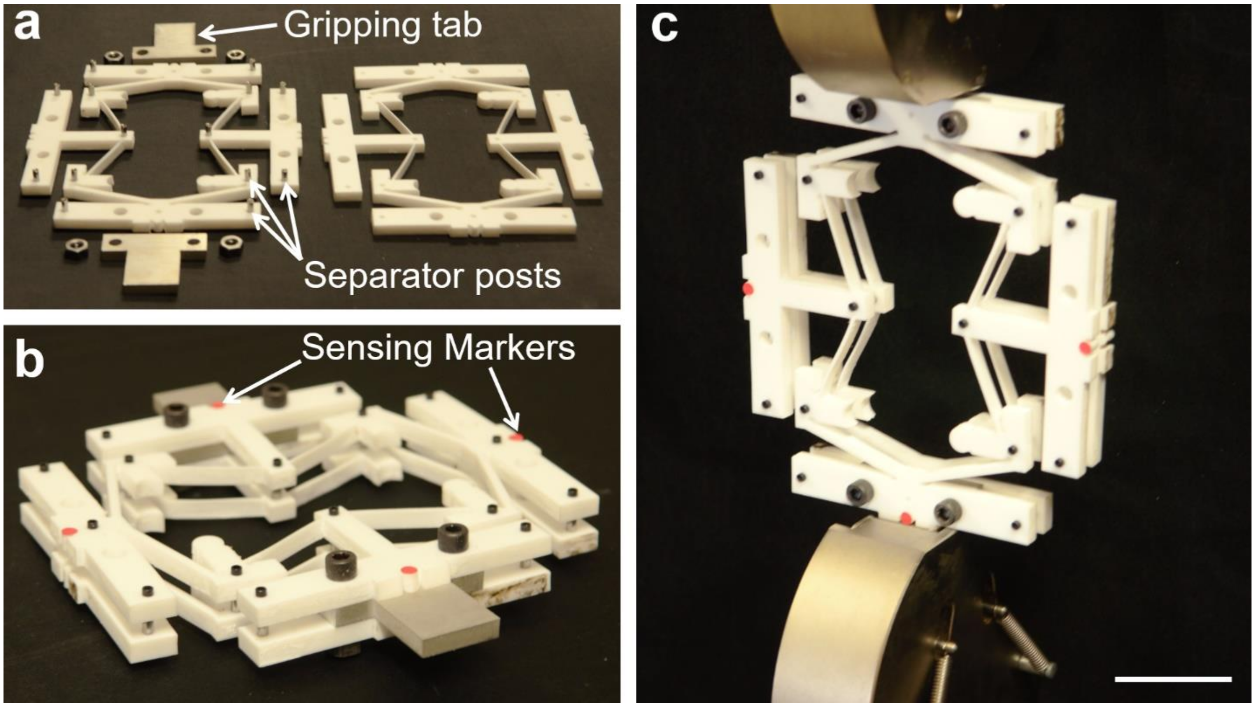

Supplementary Fig. 8 Macro-scale fabrication of individual unit cells. **a** Each cell was laser-cut from two sheets of Teflon and was then assembled together with separator posts and waterjet aluminum gripping tabs. **b** Sensing markers were used to detect how the cell's side tabs move in response to vertical strain loads. **c** The cells were loaded by an Instron testing machine that clamped onto their gripping tabs. Scale bar in **c**, 5 cm.

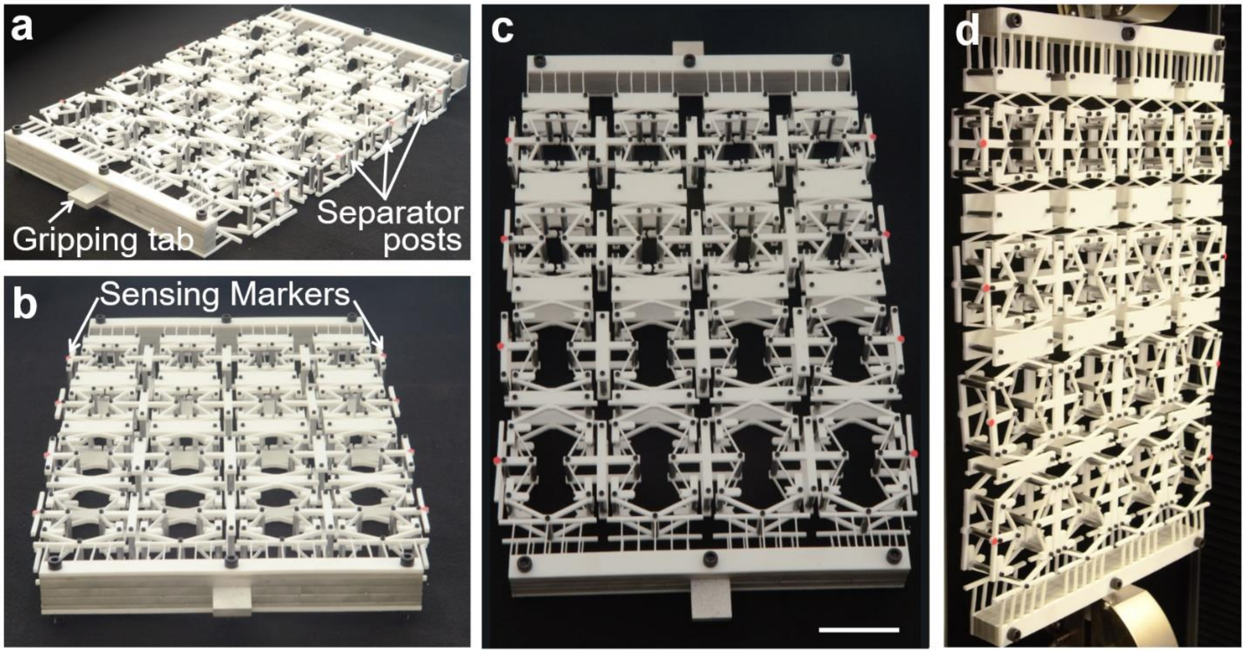

Supplementary Fig. 9 Macro-scale fabrication of a graded lattice consisting of rows of different unit cell designs. **a** Each cell was laser cut from two sheets of Teflon and was then assembled together with long separator posts and waterjet aluminum gripping tabs. **b** and **c** Sensing markers were used to detect how each row of the lattice's side tabs move in response to vertical strain loads. **d** The lattice was loaded by an Instron testing machine that clamped onto its gripping tabs. Scale bar in **c**, 5 cm.

Supplementary Tables

The supplementary tables referenced in the main text along with their corresponding legends are provided on the following pages.

Parameters	Design I	Design II	Design III
H (mm)	100	100	100
T (mm)	10	10	10
t_1 (mm)	2.1	3.7	1.5
t_2 (mm)	3.8	1.7	6
S_1 (mm)	33.5	15	31
S_2 (mm)	15	10	15
S_3 (mm)	5	5	1
S_4 (mm)	8	7	15
S_5 (mm)	15	15	15
S_6 (mm)	15	15	15
θ_1 (rad)	0.2269	0.2967	0.1396
θ_2 (rad)	0.3491	0.4014	0.4712

Supplementary Table 1. The geometric parameters of the designs named in the corresponding columns as defined according to the labels shown in Fig. 2. Images of each of the three designs are shown in Fig. 4. Italicized symbols are scalar values of geometric parameters.

Parameters	Design A	Design B	Design C	Design D
H (mm)	50	55	54	57
T (mm)	5	5	5	5
t_1 (mm)	1	1.5	2.2	2.5
t_2 (mm)	1.8	2.5	1	1
S_1 (mm)	16.8	18	7	7
S_2 (mm)	10	10	1	10
S_3 (mm)	2	1	2.5	2.5
S_4 (mm)	6	4	3.5	3.5
S_5 (mm)	4	3.5	4.5	3
S_6 (mm)	14.51397	11.13083	7.94264	2.35724
θ_1 (rad)	0.20944	0.20944	0.34907	0.47124
θ_2 (rad)	0.34907	0.57596	0.40143	0.45379

Supplementary Table 2. The geometric parameters of the designs named in the corresponding columns as defined according to the labels shown in Fig. 2. Images of each of the four designs are shown in Fig. 6a. Italicized symbols are scalar values of geometric parameters.

Parameters	3D Design Fabricated
H (µm)	100
T (µm)	10
t_1 (µm)	3
t_2 (µm)	7
S_1 (µm)	30
S_2 (µm)	0
S_3 (µm)	10
S_4 (µm)	10
S_5 (µm)	0
S_6 (µm)	1.74
S_7 (µm)	4.96
S_8 (µm)	7.9
S_9 (µm)	7.7
S_{10} (µm)	9.12
S_{11} (µm)	6.22
θ_1 (rad)	0.2269
θ_2 (rad)	0.4014

Supplementary Table 3. The geometric parameters of the 3D micro-scale unit cell design shown in Fig. 8 and Supplementary Fig. 4 as defined according to the labels shown in Fig. 2 and Supplementary Fig. 5. Italicized symbols are scalar values of geometric parameters.
