# Supplementary material for: Sequential metamaterials with alternating Poisson’s ratios
Source: Nat Commun. 2022 Feb 24;13:1041. doi: 10.1038/s41467-022-28696-9 (PMC8873317; doi:10.1038/s41467-022-28696-9)
Supplement: Supplementary file 3 — Description of Additional Supplementary Files [file 41467_2022_28696_MOESM3_ESM.pdf]

Description of Additional Supplementary Files

File Name: Supplementary Movie 1
Description: Finite element analysis (FEA) response of the Design I unit cell shown loaded from 5% to 25% strain along the y-axis in increments of 5%. Note that the FEA calculations assumed linear elastic properties of Teflon, no gravity, and lattice conditions (i.e., the cell's side tabs are constrained to not rotate as they displace to mimic the constraint that the cell would experience if it existed within an infinitely large lattice of repeating cells).

File Name: Supplementary Movie 2
Description: Finite element analysis (FEA) response of the Design II unit cell and Design III unit cell rotated 90o on their side both shown loaded with 15% strain along the y-axis. Note that the FEA calculations assumed linear elastic properties of Teflon, no gravity, and lattice conditions (i.e., the cell's side tabs are constrained to not rotate as they displace to mimic the constraint that the cell would experience if it existed within an infinitely large lattice of repeating cells).

File Name: Supplementary Movie 3
Description: An example .gif animation file that the analytical MATLAB tool of this work produces once a cell's material properties, geometric parameters, strain amplitude, and an accuracy number have been entered. The moving black line on the plot corresponds with the deformed state of the cell shown animated on the left side.

File Name: Supplementary Movie 4
Description: The finite element analysis (FEA) simulated response alongside the experimentally measured response of the Design I and Design II unit cells shown loaded with 15% strain along the y-axis. Note that the FEA calculations assumed nonlinear elastic-plastic properties of Teflon (shown in Supplementary Fig. 2), with gravity pulling in the negative y-axis direction, and without lattice conditions (i.e., the cell's side tabs are allowed to rotate).

File Name: Supplementary Movie 5
Description: The finite element analysis (FEA) simulated response alongside the experimentally measured response of the Design I and Design II unit cells rotated 90o on their sides shown loaded with 15% strain along the y-axis. Note that the FEA calculations assumed nonlinear elastic-plastic properties of Teflon (shown in Supplementary Fig. 2), with gravity pulling in the negative y-axis direction, and without lattice conditions (i.e., the cell's side tabs are allowed rotate).

File Name: Supplementary Movie 6
Description: The finite element analysis (FEA) simulated response alongside the experimentally measured response of the graded lattice of Figure 5b shown loaded with 13% strain in tension and 7% strain in compression along the y-axis. Note that the FEA calculations assumed nonlinear elastic-plastic properties of Teflon (shown in Supplementary Fig. 2) with gravity pulling in the negative y-axis direction.

File Name: Supplementary Movie 7
Description: In-situ scanning electron microscope (SEM) video of the microfabricated 3D unit cell design of Supplementary Fig. 4 with the geometric parameters specified in Table S3 being loaded alongside its corresponding strain-versus-time plot.

File Name: Supplementary Movie 8
Description: In-situ scanning electron microscope (SEM) video of a 3x3x3 lattice consisting of the microfabricated 3D unit cell design of Supplementary Fig. 4 with the geometric parameters specified in Table S3 being loaded alongside its corresponding strain-versus-time plot.

File Name: Supplementary Software 1
Description: MATLAB code that enables users to rapidly calculate and visualize the large-deformation Poisson's-Ratio behavior of any geometric version of the metamaterial design introduced.
