# Supplementary material for: Sequential metamaterials with alternating Poisson’s ratios
Source: Nat Commun. 2022 Feb 24;13:1041. doi: 10.1038/s41467-022-28696-9 (PMC8873317; doi:10.1038/s41467-022-28696-9)
Supplement: Supplementary file 12 — Supplementary Software 1 [file 41467_2022_28696_MOESM12_ESM.zip › MATLAB TOOL-Final/Readme.pdf]

System Requirements

• MATLAB R2014b or later

The package was tested on MATLAB R2021b that is installed on Windows 10 Home, version 21H2.

Installation Guide

1- Download the files from https://github.com/aminfno/Metamaterial
2- Make sure all downloaded files (‘Metamaterial.m’, ’ Metamaterial.fig’, ’Metamaterial_results.m’, ’ Metamaterial_results.fig’, ’ data.mat’) are in the same folder.

Instructions and Demo

1- Run ‘Metamaterial.m’ on MATLAB, Metamaterial window opens.
2- Change parameter values based on your design.
3- To run the simulation, press ‘Click here to run the simulation’ and wait until Metamaterial_results window opens.
    a. Output: This window contains the animation of your design sinusoidally loaded in addition to the Strain and Poisson’s ratio vs time plot.
    b. Run time: Typical run time with the preset values is about a minute. This may vary based on the parameters you choose for your design specially ‘Accuracy’ value.
4- To save the results as a GIF file, press ‘Click to save as .gif file’.
5- To return to the main window and make a new design, press ‘Go back to redesign’.
